# Demographics of Patients Undergoing Carpal Tunnel Release in an Urban Tertiary Hospital in Malaysia

**DOI:** 10.5704/MOJ.1911.009

**Published:** 2019-11

**Authors:** AS Bahar-Moni, S Abdullah, H Fauzi, SY Chee-Yuen, FZ Abdul-Razak, J Sapuan

**Affiliations:** Department of Orthopaedics Surgery, Universiti Sains Malaysia, Kepala Batas, Malaysia; * Department of Orthopaedics and Traumatology, Universiti Kebangsaan Malaysia, Kuala Lumpur, Malaysia; ** Department of Orthopaedics, Widad University College, Kuantan, Malaysia

**Keywords:** carpal tunnel syndrome, median nerve, nerve compression, tinel sign, thenar wasting

## Abstract

**Introduction:** Carpal tunnel syndrome (CTS) is the most commonly encountered neuropathy. The entrapment of the median nerve at the wrist can be corrected with a carpal tunnel release (CTR) procedure. The objective of this retrospective study was to determine the demographic, medical, and surgical characteristics of the patients with CTS who presented for CTR surgery in a tertiary hospital in Malaysia.

**Materials and Methods:** Malaysians patients with CTS who had undergone a CTR during the period from 1st June 2017 to 31st December 2017 were enrolled into the study. Each patient had a minimum follow-up of three months. The demographic data of age, gender, race and occupation, and the comorbid illnesses and associated risk factors were recorded. The prevalence and occurrence of CTS in the dominant or non-dominant hand and the effectiveness of surgical intervention were also noted. Data was collected, analysed and stored in Microsoft Excel and SPSS 25.

**Results:** There was a total of 76 cases of CTR surgeries done in 62 patients in the study. Eighty percent of the patients were female, and most of the patients belonged to the age group of 41-60 years. Malays constituted 74.2% of the patients, and 34% were housewives. Hypertension, dyslipidaemia and diabetes mellitus were the three major comorbidities. Cervical spondylosis was seen in one-fourth of the patients. Bilateral hand involvement was present in 54.8% of patients. 59.7% of CTR surgery was done on the dominant hand alone, 17.7 % CTR on the non-dominant hand alone and 22.6% CTR on both hands. Numbness and pain (50%) were the predominant presenting symptoms. The most positive signs were the Durkan test (77.6%), followed by the Tinel sign at the carpal tunnel and the Phalen’s test. At follow-up, three months or more, after the surgery, 75% of the patients showed a satisfactory improvement.

**Conclusion:** Patients, who had undergone CTR, had a higher prevalence of pre-morbid conditions, and a quarter of them presented with associated cervical spondylosis. The most common presentation was a combination of numbness and pain. Many obtained satisfactory improvement post-surgery and thus open surgery could be considered a reliable treatment for CTS.

## Introduction

Carpal tunnel syndrome (CTS) was the most commonly encountered neuropathy with entrapment of the median nerve at the wrist^1^. One in five ambulatory clinic visits was for CTS, with a high incidence and prevalence not only in the United States but in other countries as well^2^. The lifetime risk was estimated to be 10%^2^. The demographic characteristics of patients with CTS were first described by Phalen, and many subsequent series had similar findings with markedly female preponderance and a peak incidence at about 55 to 60 years of age^3^. Several medical conditions were associated with CTS, including diabetes mellitus (DM), obesity, pregnancy, contraceptives, thyroid dysfunction and inflammatory conditions such as rheumatoid arthritis^4^. CTS was a condition as much related to occupation as to age, and gender.

The classic symptoms of CTS were pain and numbness, and a tingling sensation in the distribution of the median nerve, the first three fingers and radial half of the 4th finger, with commonly an exacerbation at night. Diagnosis of CTS was a clinical one, and no single test could confirm the diagnosis. However, the nerve conduction study (NCS) was a good diagnostic test with a high degree of sensitivity and specificity, which was especially useful in patients with a doubtful presentation and as an investigation to rule out other differential diagnoses^5^.

The management of CTS could be divided into conservative and surgical. Conservative treatment included splinting, activity modification, non-steroidal anti-inflammatory drugs (NSAIDs), pyridoxine, and local injection of corticosteroids. Also, yoga, chiropractic therapy, ultrasound and laser treatments had been advocated. Conservative management could be the sole treatment or as a supportive treatment for patients waiting for surgery. Carpal tunnel release (CTR) was the surgical decompression of the entrapped median nerve, either as open or endoscopic surgery.

The purpose of this retrospective study was to determine the demographic data of age, gender, race and occupation; the prevalence of CTS in dominant or nondominant hand; as well as the comorbid illnesses or associated conditions in a subset of CTS patients treated with elective CTR surgery in a Universiti Kebangsaan Malaysia Medical Centre (UKMMC), Kuala Lumpur, Malaysia. These patients generally had a chronic condition, with more severe pathology, yet with more likely an accurate and precise diagnosis^6^.

The duration of the symptoms of CTS before presentation to the orthopaedic clinic, the length of the conservative management used before surgical decompression, the effectiveness of the open CTR, the association between the outcome of the surgery with a positive Tinel sign and the presence or absence of thenar wasting were also recorded.

## Materials and Methods

Patients with CTS who had undergone open CTR during the period from 1st June 2017 to 31st December 2017 were enrolled. Each patient had a minimum follow-up of three months. Patients with incomplete case notes, non-Malaysian patients and patients with no follow-up were excluded from the study. Data was collected from the files, operative notes and follow-up charts of the patients by medical graduates under the supervision of a specialist hand surgeon. Demographic data of age, gender, race, and occupation, along with the co-morbid and associated conditions of DM, hypertension (HTN), dyslipidaemia, thyroid and other endocrine disorder, and inflammatory joint diseases were recorded. Symptoms and signs of the CTS: hand dominance and hand affliction; duration of the disease before presentation to the hand clinic; and cervical spondylosis before the onset of CTS were recorded. Investigations consisting mainly of the nerve conduction study (NCS), and treatment options along with the duration of conservative management and the date of the open CTR and the follow-up after three months or more, together with the documentation of the effectiveness of the surgery were noted.

The effectiveness of surgical decompression was grouped as a satisfactory or as an unsatisfactory outcome. If there was an improvement of more than 50% of the presenting signs and symptoms, it was recorded as a satisfactory. An improvement of signs and symptoms less than 50% was considered as an unsatisfactory result. The association between a positive Tinel sign and the presence of thenar wasting with the outcome was derived by using the chi-square test. P <0.05 was regarded as statistically significant.

Microsoft Excel and SPSS 25 were used to analyse, and store data and results were presented in the form of descriptive statistics and tables.

## Results

During this period of the study, there were 66 patients with a total of 80 CTR surgeries done in the medical centre. Sixty-two patients with 76 cases of CTR surgeries, were enrolled in this study based on the inclusion criteria. Two patients were excluded due to incomplete follow-up notes, and another two were excluded as they were non-Malaysian. The age range of the patients was from 37 to 89 years; the mean age was 53.2 years; and the age group with the highest prevalence was 41-60 years (47%). The detailed age distribution was shown in Table I. Most of the patients were female, with a female to male ratio of 4:1. Malays comprised 74.2% of the patients, followed by Chinese (17.7%) and Indian (4.8%). 34% of patients were housewives, 21% were clerks, and 17.7% were teachers (Table II).

**Table I:** Demographic characteristics of CTS patients

DEMOGRAPHY	GROUPS	FREQUENCY	(N)	PERCENTAGE (%)
Age	18-40	15		24.2
	41-60	29		46.8
	61-90	18		29.0
Sex	Male	12		19.4
	Female	50		80.6
Race	Malay	46		74.2
	Chinese	11		17.7
	Indian	3		4.8
	Others	2		3.2

**Table II:** Occupations of CTS patients

OCCUPATION	FREQUENCY	(N)	PERCENTAGE (%)
Housewife	21		33.9
Clerk	13		21.0
Teacher	11		17.7
Medical workers	5		8.1
Manual workers	5		8.1
Total	62		100

Fifty-six patients (90%) were right hand dominant. Thirty-four (54.8%) patients presented with bilateral CTS. Among them, CTR surgery was done in both hands in 14 (22.6%) patients, according to the indication for the surgery. In the other 20 patients, one hand was treated surgically and the other hand conservatively. In total, 59.7% CTR surgery was done on the dominant hand alone, 17.7% CTR on the non-dominant hand alone and 22.6% CTR on both hands.

The patients experienced several symptoms within this subset of CTS: symptoms of tingling and numbness or in combination with pain or weakness or with both. The pain was present in 54% of the cases and weakness in 12%. A combination of numbness and pain was the commonest symptom at presentation (50%), followed by numbness alone (35.5%) (Table III). Most of the cases (93.4%) had the symptoms of CTS for less than one year.

**Table III:** Frequency of the symptoms

SYMPTOMS	FREQUENCY	(N)	PERCENTAGE (%)
Numbness and pain	38		50
Numbness alone	27		35.5
Numbness and weakness	7		9
Numbness, pain and weakness	1		1.3
Pain alone	2		2.6
Weakness alone	1		1.3

In the study, 56.5% of patients presented with one or more co-morbidities. DM was noted in 29% of patients, hypertension in 40.3%, dyslipidaemia in 27.4% and thyroid disorder in 3.2% of patients. History of steroid ingestion was noted in one patient. Associated cervical spondylosis was recorded in 22.5% of patients.

Among the 76 cases, Phalen’s test was positive in 48 (63.1%) cases, Tinel sign was positive in 52 (68.4%) cases, and Durkan test was positive in 59 cases (77.6%) cases. In 29 (38%) cases, thenar muscle atrophy was noted, whereas 47 cases (61.8%) showed a normal thenar eminence. The power of the opponens pollicis was recorded in 60 cases, and abductor pollicis brevis (APB) power was recorded in 18 cases only. Opposition was found weak in 53 cases, and APB was weak in 9 cases. All three provocative tests were found positive in 34 (44.7%) hands, and two tests were positive in 23 (30.3%) hands, one test was positive in 12 (5.8%) hands. No provocative test was positive in seven (9%) hands. All these seven hands had either thenar muscle wasting or weak opposition or APB weakness. NCS was done in 39 (51.3%) cases for assistance in diagnosis. Positive results were obtained in all of them (100%), among them, 37 cases were recorded as a moderate grade, and two were graded as severe.

All these cases were treated conservatively with vitamin B6, analgesics, splintage, physiotherapy, and occupational modification while waiting for the surgery. Among them, 72.3% of cases had received conservative management for less than six months, and 22.4% of cases had received conservative management for six to 12 months. However, conservative management protocol was not well recorded. Subsequently, these patients received surgical intervention.

Among the 76 cases of CTR, all except one was found with, at least some improvement of their previous signs and symptoms. Fifty-seven cases (75.0%) obtained a satisfactory outcome but in 18 cases (23.7%), there was an improvement of less than 50% of the symptoms which was regarded as an unsatisfactory outcome.

Among the 18 patients with DM, bilateral CTR was done in four patients. Out of these 22 cases of CTR, 19 cases were recorded with a satisfactory outcome. Among the two patients, having associated thyroid disorders, one reported a satisfactory outcome. In 14 patients with cervical spondylosis, 18 CTR surgeries were done, and only 12 cases were reported with a satisfactory outcome. The patient who reported no improvement at all following CTR surgery also had an associated cervical spondylosis (Table IV).

**Table IV:** Effectiveness of CTR surgery in the presence of co-morbidities

Co-morbidities			Satisfactory		Unsatisfactory	
			Frequency(N)	Percentage(%)	Frequency(N)	Percentage(%)
DM	Present	22	19	86.3	3	13.7
	Absent	54	38	70.3	16	29.7
Endocrine Disorder	Present	2	1	50	1	50
	Absent	74	56	75.6	18	24.4
Cervical Spondylosis	Present	18	12	66.6	6	33.3
	Absent	58	45	77.5	13	22.5

Out of the 76 cases of CTS, 52 cases (68.4%) were presented with a positive Tinel sign. 40 cases (52.6%) had satisfactory improvement, and 12 (15.8%) had unsatisfactory results. Among 24 (31.6%) Tinel negative cases, 17 (22.4%) had satisfactory, and 7 (9.2%) had unsatisfactory results. There was no statistically significant association between the positive Tinel sign and a satisfactory outcome (Table V). Twenty-nine (38.2%) cases presented with thenar muscle wasting. Among them, twenty (26.3%) had a satisfactory improvement, and in nine (11.9%), results were unsatisfactory. Among 47 (61.8%) normal thenar muscle cases, 37 (48.7%) presented with a satisfactory result and 10 (13.1%) cases reported unsatisfactory results. There was also no statistically significant association between thenar wasting and a satisfactory outcome. In the unsatisfactory group, there was one patient, who presented with no improvement at all following surgery. He belonged to the Tinel sign negative and the thenar wasting group.

**Table v:** Association between Tinel sign and Outcome of surgery The outcome of surgery - Tinel sign Crosstabulation Count

		Tinel sign		Total
		positive	negative	
Outcome of surgery	Non-satisfactory	12	7	19
	satisfactory	40	17	57
Total		52	24	76

**Table:** Chi-Square Tests

	Value	df	Asymp. Sig. (2-sided)	Exact Sig. (2-sided)	Exact Sig. (1-sided)
Pearson Chi-Square	.325^a^	1	.569		
Continuity Correction^b^	.081	1	.776		
Likelihood Ratio	.319	1	.572		
Fisher's Exact Test				.580	.382
Linear-by-Linear Association	.321	1	.571		
N of Valid Cases	76				

*a. 0 cells (0.0%) have expected count less than 5. The minimum expected count is 6.00.

*b. Computed only for a 2x2 table

## Discussion

CTS is a common condition with potentially high social and economic costs, especially if surgical treatment is required^7^. Gelfman *et al*, found a prevalence of CTS highest in the age group of 50-59 years followed by the age group of 41-49 years for the females, and for the males, the prevalence was more in the age group of 70-79 years^8^. Though the Malaysian study was conducted on a subset of CTS with a surgical release, the presentation with CTS was also highest (46.8%) in a similar age group of 41-60 years. The reported incidence of CTS was more in females^3, 9^, as in this study, where 80.6% of the patients were females, and similar with the study by Bahou, where 84.32% of the patients with CTS were female^9^. Zhang *et al* observed that patients in the CTR group were more likely older and female and with higher body mass index (BMI)^6^. CTS in women was higher than in men due in part to differences in carpal tunnel volume between men and women.

In this study, the respondents were mainly Malays (74.2%), followed by Chinese (17.7%) and Indians (4.8%) reflecting the different ethnic composition as reported in the Malaysia Population Census 2010, where the Bumiputera (Malay) comprised (67.4%), Chinese (24.6%), Indians (7.3%) of the population of Malaysia^10^.

The work-related issues of CTS are still debatable. Except for the significant exposure to vibration. High prevalence of CTS in some specific occupations. It was generally accepted that work was just one component of the many factors that contributed to, and aggravated compression neuropathy. The study showed that housewives (33.9%) had the highest prevalence of CTS, with many affected in the perimenopausal age group. A likely cause of the high incidence of CTS among housewives could be the physiological changes of menopause that might render the nerve susceptible to compression.

Workers who used their hands and wrists repetitively were usually at risk for CTS. The clerks (21%), teachers (17.7%), health care workers (8.1%) and manual workers were also more affected with CTS. The high prevalence of CTS among health care workers could also be due to a higher awareness of health issue as compared to those in other occupations. Also, there were two patients, one who worked as a grocery worker and the other as a chef. All were in occupations that shared similar characteristics, with repetitive tasks of hand involving (flexion and extension) and with prolonged duration of exposure^11^.

The common pre-morbid conditions that were associated with CTS were DM, dyslipidaemia, thyroid disorder, inflammatory arthritis, smoking, use of steroids, and a fracture of the wrist. 56.5% of the patients had some underlying pre-morbid conditions. Hypertension was the highest (40.3%), followed by the presence of DM (29%), and dyslipidaemia (27.4%). 3.2% of patients had an underlying thyroid disorder. Several studies concluded that the patient with DM, dyslipidaemia or metabolic syndrome had a higher prevalence of CTS1,^11, 12^. Steroid usage was also a significant risk factor for CTS, and one of the patients was on long term steroid.

Upton *et al* attributed double-crush mechanism as an important concept in nerve compression. In their clinical review of 115 patients with median or ulnar nerve compression, 81 patients had evidence of a cervical root lesion^13^. They stated that compression of a nerve at one level would make the nerve more susceptible to damage at another level^13^. In the study, 22.5% of the patients in the study had associated cervical spondylosis.

90.3% of the patients were right-handed, and in most of the cases (82.3%), the dominant hand was affected alone or along with its non-dominant counterpart. This result was consistent with the study by Bagatur *et al*, where they suggested that CTS was a bilateral disorder and showed that the dominant hand involvement was more^14^.

There were several symptoms experienced by patients with CTS. The commonest was numbness (96.1%) alone or in combination with pain and weakness. Numbness and pain were the commonest combinations of presenting symptoms (50%), followed by numbness alone (35.5%) and, numbness plus weakness (9%). Several studies mentioned that the most characteristic manifestation of CTS was paresthesia and pain2.

The Durkan test was positive in most of the cases (77.6%) followed by the Tinel sign (68.4%), and the Phalen’s test (63.1%). Gerr and Letz stated that the sensitivity for the Phalen’s test ranged from 10% to 88% and of the Tinel sign from 26% to 79%^15^. The median nerve entrapment in the carpal tunnel caused thenar muscle weakness. Moreover, prolonged disuse could lead to atrophy and wasting. In the study, thenar muscle wasting was recorded in 38.2% cases.

In this study, 51.3% of the cases had an NCS, and all were positive for CTS and were of either moderate or severe grade. Jablecki *et al* stated that NCS was a definitive diagnostic test for CTS with a high degree of sensitivity and specificity which could exclude other peripheral conditions with similar symptoms5. In 48.7% of cases, NCS was not done before CTR where the clinical signs and tests were strongly positive.

In this study, all the cases received conservative management while waiting for the surgery. None of the patients was given a steroid injection. Dammers *et al* conducted a randomised, double-blind, placebo-controlled trial to assess the efficacy of 40mg of methylprednisone injection proximal to the carpal tunnel and stated that a single injection of steroids close to the carpal tunnel could result in long term improvement and should be considered before surgery^16^. They also stated that one injection of methylprednisolone could delay the need for carpal tunnel surgery for up to one year^16^.

Among 34 patients, with bilateral CTS, surgical decompression was done in 48 hands. In 14 patients, CTR was done on both hands, in 14 cases only on the right hand, and in 6 cases on the left hand only following indications for the surgery. Bagatur *et al* treated the more severely affected hand first even when bilateral symptoms were present14. And out of 77 patients, who had bilateral CTS, 32 respondents (42%) had unilateral CTR done despite bilateral problems^14^.

The open CTR was an effective treatment of CTS, as all the patients, but one, reported some improvement of symptoms following CTR and 75.0% of the patients reported a satisfactory outcome. Thurston *et al* found that more than 80% of his patients had complete relief of, or a marked improvement in, the symptoms after open CTR. His patients were assessed at a minimum follow-up of 18 months^17^. Zamborsky *et al* proved the surgical approach to be more efficient than the conservative method of steroid injection and splinting^18^. In this study, the post-surgery follow-up period was three to nine months as the attendance in the follow-up clinic was irregular after three months. The minimum follow-up period of three months was not long enough for all CTR cases. Because the sensory and motor reinnervation in severe cases might take longer time to recover and that might be a cause of the 25% unsatisfactory outcome following CTR. Unsatisfactory results were most probably attributed to surgical complications like nerve injury during release, infection, haemorrhage from accessory median artery or to incorrect pre-operative diagnosis like radiculopathy of the C7-root, cervical myelopathy or polyneuropathy.

Complications were recorded in only two (2.6%) cases. In one case there was a post-operative infection, with initial improvement of the symptoms following CTR but with recurrence of the symptoms soon after. In the second case, there was a recurrence of the symptoms after 12 months. Both patients were treated conservatively after that. Information regarding pillar pain and post-CTR grip strength was not well recorded.

## Conclusion

In this study, the patients, who had undergone CTR, had a higher prevalence of pre-morbid conditions, with associated cervical spondylosis in a quarter of them. The most common presentation was a combination of numbness and pain. Despite the limitations of a retrospective study with a short follow-up period, most of the patients obtained satisfactory improvement post-surgery. Open surgery could thus be considered as a reliable treatment for CTS.
